# Multi-organ FGF21-FGFR1 signaling in metabolic health and disease

**DOI:** 10.3389/fcvm.2022.962561

**Published:** 2022-08-02

**Authors:** Namrita Kaur, Sanskruti Ravindra Gare, Jiahan Shen, Rida Raja, Oveena Fonseka, Wei Liu

**Affiliations:** Division of Cardiovascular Sciences, School of Medical Sciences, Faculty of Biology, Medicine, and Health, The University of Manchester, Manchester, United Kingdom

**Keywords:** diabetes mellitus, metabolic stress, multi-organ signaling, treatment, heart failure

## Abstract

Metabolic syndrome is a chronic systemic disease that is particularly manifested by obesity, diabetes, and hypertension, affecting multiple organs. The increasing prevalence of metabolic syndrome poses a threat to public health due to its complications, such as liver dysfunction and cardiovascular disease. Impaired adipose tissue plasticity is another factor contributing to metabolic syndrome. Emerging evidence demonstrates that fibroblast growth factors (FGFs) are critical players in organ crosstalk *via* binding to specific FGF receptors (FGFRs) and their co-receptors. FGFRs activation modulates intracellular responses in various cell types under metabolic stress. FGF21, in particular is considered as the key regulator for mediating systemic metabolic effects by binding to receptors FGFR1, FGFR3, and FGFR4. The complex of FGFR1 and beta Klotho (β-KL) facilitates endocrine and paracrine communication networks that physiologically regulate global metabolism. This review will discuss FGF21-mediated FGFR1/β-KL signaling pathways in the liver, adipose, and cardiovascular systems, as well as how this signaling is involved in the interplay of these organs during the metabolic syndrome. Furthermore, the clinical implications and therapeutic strategies for preventing metabolic syndrome and its complications by targeting FGFR1/β-KL are also discussed.

## Introduction

FGFR (Fibroblast Growth Factor Receptor) signaling is involved in various stages of human development and metabolic health. In humans, there are 23 distinct fibroblast growth factors (FGFs), 18 of which (FGF1-10 and 16-23) are mitogenic signaling molecules that bind to four high-affinity cell surface receptors, named FGFR1, FGFR2, FGFR3, and FGFR4 (1). The ligand-binding affinity and tissue distribution of these receptors differ across organs (2). FGFR1 is found in a wide range of cell types and tissues and is located on chromosome 8 at position 11.23 in humans (1, 2). Structurally, FGFRs are single-transmembrane proteins that consist of an extracellular ligand-binding domain and a split functional intracellular kinase domain (1). The intracellular domain is responsible for FGFR tyrosine kinase activity, along with phosphorylation or autophosphorylation of the receptor molecule (3). Studies have shown that the binding of FGFs and FGFRs on the cell membrane induces a variety of biological responses, such as stimulating the formation of new blood vessels, promoting the development and differentiation of embryonic tissues, participating in wound healing and tissue regeneration, neurotrophy and regulation of endocrine effects (3, 4).

Among the FGF family, FGF19, FGF21, and FGF23 act as endocrine hormones that diffuse into circulation to operate on distal tissues (4). Particularly, FGF21 is expressed in numerous organs and is a key regulator in the body upon metabolic or environmental stresses, such as fasting, food overload, autophagy insufficiency, oxidative stress and exercise (5). FGF21 has significant impacts and potential therapeutic applications in several metabolically active tissue organs, including the heart, liver and adipose tissue which are discussed in detail further. Emerging experimental studies highlight the metabolic effects of FGF21 in maintenance of energy homeostasis, glucose and lipid metabolism, and insulin sensitivity (6–8). In addition, FGFRs are diverse in their subtypes and functions. Thus, endocrine FGF21 not only binds to FGFR1 but also with the obligatory co-receptor βeta-Klotho (β-KL) for signaling specificity (9, 10). FGF21-FGFR1/β-KL signaling is therefore involved in a variety of biological functions, including pro-survival signals, anti-apoptotic signals, and cell proliferation and migration stimulation (11, 12). This review discusses the current understanding of the role of FGF21-FGFR1/β-KL signaling pathway across multiple metabolic organs under metabolic health and disease.

## FGF21-FGFR1 signaling in liver

FGF21, along with β-KL is upregulated in the liver by nutritional stresses like starvation, amino acid restriction, and high-fat diet (HFD) or ketogenic diets, thereby mediating hepatic response to nutritive stimuli (13–15). Moreover, acute and chronic stress including exercise, oxidative stress and liposaccharides content also increase FGF21 levels (16, 17). Another contributor of hepatic FGF21 expression is hepatic ER stress that is mediated by eukaryotic translation factor 2α-activating transcription factor 4 (eIF2α-ATF4) pathway (18). Many studies have highlighted the key role of FGF21-mediated FGFR1/β-KL activation in the regulation of hepatic lipid and glucose metabolism (15). The overexpression of hepatic FGF21 in mice showed increased ketogenesis, gluconeogenesis, and lipolysis, thereby regulating hepatic metabolism under prolonged fasting (14). Mechanistically, FGF21 induced the expression of peroxisome proliferator-activated receptor gamma coactivator 1α (PGC-1α) and improved β-oxidation of fatty acids, thereby improving adaptive starvation response in the liver in response to prolonged chronic fasting (19). Further, exogenous FGF21 treatment improved liver metabolism (19) and insulin sensitivity (20) in the obese C57BL/6 mice by inducing phosphorylation of downstream pathways, including fibroblast growth factor receptor substrate 2 alpha (FRS2α) and extracellular signal-regulated kinase (ERK) (20). These studies thus indicate the role of FGF21 in improving obesity or prolonged fasting induced metabolic stress. However, there is a low level of endogenous FGFR1 expression in the liver (21), so it is unclear whether the beneficial effects of FGF21-β-KL signaling are mediated directly through FGFR1. Also, FGF21 was shown to have no effect in isolated hepatocytes from mouse and rat (22). This results from insufficient peripheral signals from adipose tissue, modulating the liver's response to FGF21 indirectly. Therefore, a deeper understanding of what extent and how FGF21-FGFR1/β-KL signaling contributes to hepatic metabolic responses needs to be obtained.

## FGF21-FGFR1 signaling in adipose tissue

Adipocytes express both β-KL and FGFRs (mainly FGFR1 and FGFR2) and are important targets for FGFs (23). White adipose tissue (WAT) helps in storing energy, whereas brown adipose tissue (BAT) helps in energy expenditure by generating heat through a process called thermogenesis (24). FGF21 expression is induced by exposure to cold or stimulation by β-adrenergic receptors in the adipose tissue (25–28). Multiple genetic and pharmacological studies highlight the role of FGF21-FGFR1/β-KL signaling pathway in regulating adipose tissue metabolism (10, 23, 29, 30). Studies showed that long-term HFD-fed obese mice exhibited hyperglycemia, hyperinsulinemia, and hyperlipidemia, with markedly reduced FGFR1 and β-KL expression in adipose tissue (31). WAT-specific knockout of β-KL/FGFR1 reduced FGF21 response in WAT and eliminated the beneficial effects, such as weight loss and energy expenditure in the obese rodents (31). In addition, Chen et al. found that anti-FGFR1/β-KL bispecific antibody (acting as FGF21 mimetic) stimulated energy expenditure in adipocyte-selective FGFR1-deficient mice, elucidating the indirect role of FGF21 in BAT thermogenesis *via* uncoupling protein 1 (Ucp1) activation (32). Thus, BAT has gained attention as a novel target for treating obesity and Type 2 diabetes due to its “fat-burning” properties (33), mediated by FGF21-FGFR1 signaling (34, 35). BAT-derived FGF21 either functions locally or escapes into the systemic circulation, having an autocrine as well as an endocrine role in thermogenesis *via* PGC-1α, mitogen-activated protein kinase (MAPK) and ERK signaling (27, 28, 36). Moreover, prolonged treatment of FGF21 on brown adipocytes increased glucose consumption (28) and insulin-stimulated glucose uptake *via* hepatic adiponectin secretion in a paracrine manner (22, 37, 38). This suggests a hepatic-adipose crosstalk. However, two groups independently showed that surgical removal of BAT did not alter the effects of FGF21 in obese rodents (7, 39), indicating that BAT activation and WAT browning alone are not responsible for the systemic metabolic benefits of FGF21 treatment (40). Therefore, further studies, especially clinical trial with existing FGF21 analogs are needed to establish the underlying mechanisms by which FGF21-FGFR1/ β-KL signaling governs systemic metabolism in the adipose tissue.

## FGF21-FGFR1 signaling in the heart

Emerging evidence shows that FGF21-FGFR1 signaling is also an important regulator in the heart. For instance, it is stimulated *via* paracrine and endocrine FGFs and exhibits anti-hypertrophic, anti-oxidative and anti-apoptotic properties under physiological and pathological conditions (41–44). Endocrine FGF21 has been shown to have cardiovascular protective effects, specifically in ischemic/reperfusion injury (45), isoproterenol-induced cardiac hypertrophy (46), alcoholic cardiomyopathy (47), and hypertensive heart disease (48). FGF21 activity in the heart is dependent on its binding to FGFR1 and β-KL and induces cell survival *via* anti-oxidative mechanisms and recovery of energy homeostasis in cardiac cells (49). In clinics, myocardial FGF21 is increased in advanced heart failure; however, in a pre-clinical ischemic mouse heart, FGF21 induction is not apparent (43). Nevertheless, FGF21 inhibits cardiac remodeling by activating MAPK signaling in an autocrine manner (41). Following myocardial infarction, FGF21 exerts its cardioprotective action *via* ERK 1/2 and AMP-activated protein kinase (AMPK) in an acute manner and *via* Phosphoinositide 3-kinases (PI3K)/ protein kinase B (Akt) in a sustained fashion (45, 50). Of note, cardiomyocytes can also produce FGF21 in response to disturbances in cellular metabolism (51). An earlier study demonstrated that FGF21 is secreted into the culture media at a basal rate of 0.05 ng/mL per 24 h, thereby establishing FGF21 as a cardiomyokine. The cardiac FGF21 autocrine loop is likely a compensatory mechanism initiated in response to oxidative stress (52). Global FGF21 knockout results in heightened cardiomyocyte inflammatory response *via* increased nuclear factor kappa B activity and upregulation of interleukin 6, concomitant with repressed fatty acid oxidation. Moreover, hypertrophic stimuli induce transcriptional upregulation of cardiac FGF21 *via* Sirtuin 1- PPARα pathway (46). FGF21 directly affects the heart, owing to FGFR1 and β-KL expression in the myocardium (53); however, the molecular basis whereby the FGF21-FGFR1 pathway is involved in cardiac metabolism is elusive.

In streptozotocin (STZ)-induced diabetes, cardiac FGF21 mRNA level is increased significantly (54). FGF21 mediated FGFR1 activation enhanced ERK1/2 phosphorylation, p38 MAPK activity, and AMPK activation, thereby impeding diabetes-induced apoptosis (53). FGF21 global knockout mice are more likely to develop STZ-induced diabetic cardiomyopathy. This is accompanied by severe cardiac dysfunction, structural changes, oxidative stress, and cardiac lipid accumulation *via* cluster of differentiation 36 (CD36) upregulation owing to decreased lipid oxidation, and impaired glucose oxidation. Conversely, using genetic or pharmacological modulation, FGF21 displays cardioprotective properties under dysregulated glucose and lipid metabolism (55–57) FGF21 also promotes lipophagy in mouse cardiomyocytes in obesity-related cardiomyopathy by preventing lipid accumulation (58). In addition, FGF21 protects the heart against Type 2 diabetes by either AMPK-protein kinase B (PKB, also known as AKT)-nuclear factor erythroid 2-related factor 2 (NRF2)-mediated anti-oxidative pathway or acetyl-CoA carboxylase (ACC)-Carnitine palmitoyltransferase I (CPT-1) lipid-lowering pathway, primarily attributable to managing lipotoxicity (59).

Moreover, upon hyperglycemia and hyperlipidemia, endoplasmic reticulum (ER) stress is invoked by oxidative stress, lipid deposition, and abnormal proteins synthesis in cardiomyocytes (60). Maladaptive ER stress eventually disturbs lipid synthesis, calcium homeostasis, protein quality control, leading to cell death (61). FGF21 diminishes ER stress-mediated myocardial apoptosis *via* reduction of ATF4-C/EBP homologous protein (CHOP) pathway (62). Although cardiac-specific overexpression of FGF21 does not play a major role in cardiac energy metabolism under an unstressed-state, FGF21 secretion is activated upon cardiac ER stress altering cardiac glucose oxidation in an autocrine manner (63).

Additionally, FGF21 signaling exerts anti-inflammatory effects by inhibiting PI3K/AKT signaling in the diabetic heart (56) and by promoting AMPK-paraoxonase 1 axis in high-glucose stressed cardiomyocytes (64). On the other hand, FGFR1 signaling is necessary for anti-fibrosis. Endothelial FGFR1 knockout mice showed considerable kidney and heart fibrosis (65). Moreover, FGF21 has anti-oxidative properties *via* AMPK activation in endothelial cells under diabetic stress (66). Furthermore, global β-KL knockout mice show reduced serum levels of adiponectin, known to modulate FGF21 signaling in several organs. Accordingly, global adiponectin knockout mice display diminished cardioprotective effects of FGF21 (67). In general, current research points to the potential importance of further investigating cardiac FGF21-FGFR1/β-KL signaling in metabolic stress.

## Multi-organ crosstalk mediated by FGF21

**Figure 1:**
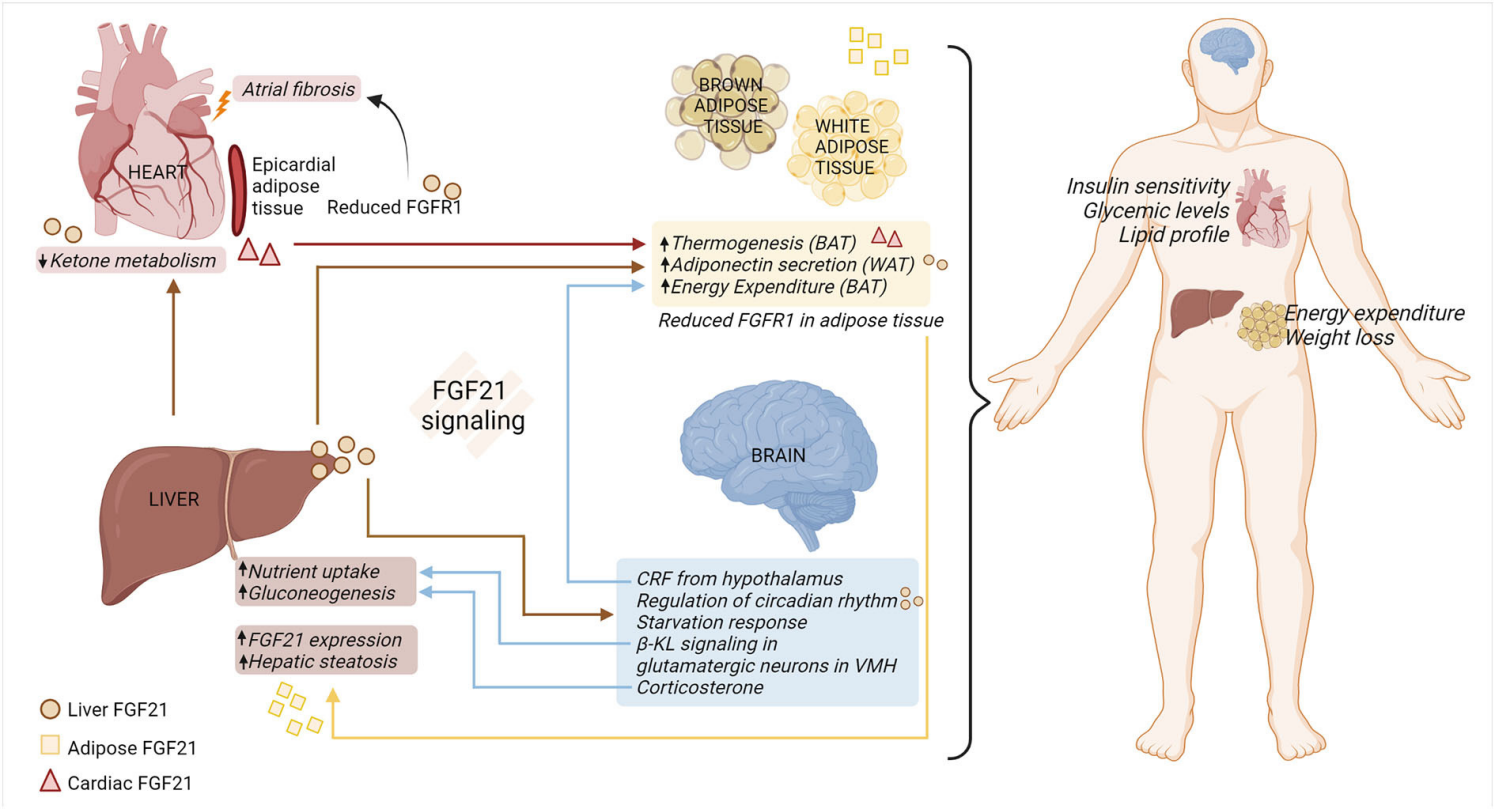
FGF21-FGFR1/β-KL regulation of multi-organ crosstalk under metabolic stress. FGF21-FGFR1/β-KL signaling is vital in regulating systemic and organ responses under pathophysiological metabolic stress. Hepatic sourced FGF21 (circle) reduces ketone metabolism in the cardiomyocytes, induces secretion of adiponectin from WAT, and modulates systemic metabolism. Moreover, hepatic sourced FGF21 passes through the blood-brain barrier and is involved in regulation of circadian rhythm and appetite response. In a feedback loop, β-KL signaling in the VMH-specific glutamatergic neurons contributes to modulating hepatic nutrient uptake. The release of CRF and corticosterone from the brain is responsible for regulating energy expenditure in the adipose tissue and liver gluconeogenesis, respectively. Cardiac sourced FGF21 (triangle) promotes thermogenesis in the adipose tissue, thus improving overall metabolic health. Under the adipo-hepatic communication, reduced expression of adipose FGFR1 aggravates hepatic steatosis and adipose FGF21 (square) increases the expression of hepatic FGF21. Hence, multi-organ crosstalk mediated by FGF21-FGFR1/β-KL signaling alleviates metabolic distress by improving insulin sensitivity, glucose, and lipid levels in the body, along with increased energy expenditure in the adipose tissue and weight loss. BAT, brown adipose tissue; β-KL, beta-klotho; CRF, corticotropin releasing factor; FGF21, fibroblast growth factor 21; FGFR1, fibroblast growth factor receptor 1; VMH, ventromedial hypothalamus; WAT, white adipose tissue (created with Biorender.com).

FGF21 response in organs appears to be influenced by tissue-specific interactions (68), summarized in Figure 1. FGF21 stimulates adiponectin secretion from adipocytes, which confers metabolic actions on the other cells/tissue, such as blood vessels (69). The effects of FGF21 are due to its direct action on hepatocytes or cardiomyocytes, and/or indirect impacts on the brain–hepatic axis. Peripheral signals, along with gastro-intestinal hormones, are responsible for conveying metabolic information to the brain and modulating glucose homeostasis and energy intake in the body (70). Although FGF21 is not expressed in the central nervous system (CNS), it can pass through the blood-brain barrier, allowing communication between peripheral tissues and the CNS (71). It was evidenced by a study utilizing β-KL^Camk2a^ mouse, that lacks β-KL in the hypothalamus and the hindbrain. This model confirmed central FGF21 signaling involved in the regulation of the circadian rhythm and starvation response (72). Additionally, β-KL- glutamatergic knockout mice elucidated that FGF21-FGFR1/β-KL signaling in the ventromedial hypothalamus decreases sucrose consumption/sweet-taste preference, eventually protecting the hepatic metabolism (73). FGF21 is also responsible for stimulating corticotropin-releasing factor and corticosterone in the brain, which subsequently participates in energy expenditure in the BAT (74–76) and hepatic gluconeogenesis, respectively (72, 77). Moreover, a large cohort study conducted by Jiao et al. found that FGFR1 protein in adipose tissue increased in the obese women, and the hypothalamic expression of FGFR1 was increased in the diet-induced obese rats (78). This study thus highlighted FGFR1 as a novel obesity gene that influences adipose tissue and the hypothalamus, thereby initiating obesity and modulating appetite, respectively.

Moreover, the hepatic-cardiac signaling circuit has been documented in human heart failure samples. This study highlights the endocrine action of hepatocyte sourced FGF21, resulting in enhanced binding of FGF21 to diseased cardiomyocytes. This increase in FGF21 binding was associated with reduced ketone metabolism in the heart (11). In addition, cardiac-sourced FGF21 modulates the metabolic phenotype of BAT by promoting thermogenesis in obese mice with cardiac muscle autophagy deficiency (12). Collectively, further research is needed to explore the role of hepatic- and/or cardiac-sourced FGF21 on FGFR1 signaling across multiple organs under metabolic stress.

Moreover, adipocyte lipolysis releases fatty acids into the bloodstream. These fatty acids subsequently enhance FGF21 expression *via* an indirect mechanism by activating PPARα in hepatocytes (79). Interestingly, one study highlighted that global β-KL knockout increases energy expenditure from BAT, making the mice resistant to obesity (80). Moreover, adipo-hepatic communication was noticed by adipocyte ablation of FGFR1. Adipocyte-specific deletion of FGFR1 aggravates hepatic steatosis (81), indicating the plausible FGFR1 regulation on maintenance of energy homeostasis across multiple organs. Finally, the browning of epicardial adipose tissue (EAT) contributes to atrial fibrillation under diabetic stress. Mechanistically, micro-RNA (miR)-21-3p is significantly upregulated in serum from diabetic patients and participates in atrial fibrosis under hyperglycemia conditions by reducing FGFR1 in EAT (82).

## FGF21 resistance in obesity and diabetes

Despite increased serum levels of FGF21 in obesity patients, no metabolic benefits were observed. Therefore, the term “FGF21 resistance” was examined in animal studies, showing reduced FGFR1 and β-KL in adipose tissue in obese mice (83). FGF21 effects on insulin sensitivity is then impeded (84). In addition, FGF21 resistance was also observed post FGF21 administration in obese mice (85). Of note, regarding the role of expression of β-KL in FGF21 resistance in adipose tissue, different results have been reported in obese mice. Although β-KL reduction is not associated with FGF21 resistance (86), its overexpression enhances FGF21 action in adipocytes (87). Additionally, β-KL has been shown to be an integral part of the FGF21 machinery in the liver. In the mice lacking β-KL, FGF21 was defective in regulating lipid and glucose metabolism at the whole organism level in diet-induced obesity (30). Thus, further preclinical and clinical studies are required to determine the molecular basis of FGF21 resistance, particularly in distinct cells.

Recently, serum FGF21 levels were associated with diastolic cardiac dysfunction in humans with cardiovascular diseases, such as dyslipidemic patients with coronary artery disease (50), but only a few reports have examined FGF21's role in heart failure (88). Pre-clinical model shows that FGF21 resistance is likely involved in the impairment of glucose uptake in heart (50). Although there was no discernible difference in FGFR1 levels in hearts from obese and lean rat, β-KL was less expressed in the heart, possibly explaining FGF21 resistance (50). However, exploration of molecular basis and targeting potential of FGF21 resistance in heart is needed for therapeutic implications of heart failure.

## Targeting FGF21-FGFR1/β-KL signaling to tackle metabolic stress

It is acknowledged that targeting the FGF21-FGFR1 signaling pathway is advantageous for tackling metabolic stress. Of note, there is an increase in circulating levels of fibroblast activation protein alpha (FAP), a prolyl peptidase related to the dipeptidyl peptidase IV (DPP-IV) enzyme. Increased circulating FAP levels are associated with decreased levels of bioactive to total FGF21, thus impairing its metabolic regulation potential (89). Hence, using long-lasting FGF21 analogs and targeting FGFR1 signaling to combat resistance in several organs could be advantageous. However, the tissue specific effects have not yet been investigated in detail. FGF21 analogs are reported to adjust systemic metabolism in obese and diabetes in clinical trials and pre-clinical studies. For instance, LY2405319, improved dyslipidemia in obese patients with Type 2 diabetes (90) and diabetic monkeys (91, 92). Recently, AKR-001, an Fc-FGF21 analog, also showed beneficial effects on insulin sensitivity and lipoprotein profile in Type 2 diabetes patients (93).

Because pharmacokinetic properties of FGF21 analogs remain the most challenging for balancing therapeutic benefits and mechanism-related toxicity, further research on targeting FGFR1/β-KL signaling is crucial to identify novel therapeutic potentials (94). For instance, endocrine FGF23 bears structural similarity to FGF21 and FGF23 C-terminal alteration to FGF21 C-terminal enhances the ability of scaffold forming of FGF21-like molecule to FGFR1/β-KL complex (95). In addition, one bi-specific avimer for the complex of FGFR1 and β-KL, C3201, improves insulin sensitivity and lipid profiles in male obese cynomolgus monkeys (96). Of note, the FGFR1c/β-KL bispecific antibody, BFKB8488A, demonstrated sustained improvements in cardio-metabolism and weight loss, despite that insulin sensitivity was not consistently improved and lipoprotein responses varied in obese humans (97). However, it is necessary to further investigate the tissue-specific effects of the above-mentioned agents, including on cardiac, liver and adipose tissue function.

## Conclusion

FGF21 is an endocrine and cell-autonomous autocrine regulator displaying a varied response across different organs in a stress- and time-dependent manner (43, 98). Most studies have focused on hepatic sourced FGF21 (endocrine action) in the past. However, adipose- and cardiac muscle-sourced FGF21 require further attention to delineate their paracrine and/or autocrine roles in metabolic diseases. In addition, downstream effectors of the FGF21-FGFR1 signaling cascade in distinct cells also require further investigation. Moreover, the molecular basis underlying FGF21 resistance in organs is undocumented. Finally, the lack of improvement in insulin sensitivity in humans, despite the beneficial effects of FGF21 analogs, necessitates the development of novel therapeutic approaches targeting FGFR1/β-KL signaling in metabolic organs.

## Author contributions

NK, SG, and JS collected references, generated, drafted, did revisions, and proofread the manuscript. NK and RR generated the figure. OF proofread the manuscript. NK and WL designed the manuscript. All authors contributed to the article and approved the submitted version.

## Funding

This work was supported by grants FS/15/16/31477, FS/18/73/33973, PG/19/66/34600, and FS/19/70/34650 to WL from the British Heart Foundation.

## Conflict of interest

The authors declare that the research was conducted in the absence of any commercial or financial relationships that could be construed as a potential conflict of interest.

## Publisher's note

All claims expressed in this article are solely those of the authors and do not necessarily represent those of their affiliated organizations, or those of the publisher, the editors and the reviewers. Any product that may be evaluated in this article, or claim that may be made by its manufacturer, is not guaranteed or endorsed by the publisher.
